# Kidney Stone Prevention: Is There a Role for Complementary and Alternative Medicine?

**DOI:** 10.3390/nu15040877

**Published:** 2023-02-09

**Authors:** Adamasco Cupisti, Domenico Giannese, Claudia D’Alessandro, Alessia Benedetti, Vincenzo Panichi, Carlo Alfieri, Giuseppe Castellano, Piergiorgio Messa

**Affiliations:** 1 Department of Clinical and Experimental Medicine, University of Pisa, 56126 Pisa, Italy; 2 Nephrology, Dialysis and Renal Transplantation, Fondazione IRCCS Ca’ Granda Ospedale Maggiore Policlinico, 20162 Milan, Italy; 3 Department of Clinical Sciences and Community Health, University of Milan, 20122 Milan, Italy

**Keywords:** complementary and alternative medicine, kidney stone, urolithiasis, diet, dietary supplement, herbal products

## Abstract

Complementary and alternative medicine (CAM) is often implemented in kidney stone patients. It consists of preparations including different ingredients, such as herbs, probiotics, and vitamins, often together with alkali, that are classified within the dietary supplementation category. The majority of dietary supplements claiming to treat or prevent kidney stones contain ingredients with conflicting or no scientific evidence to support their claims. Clinicians should advise stone formers that the effects of most supplements are unknown or unstudied in humans and that the absence of evidence does not imply absence of potential harm. Unfortunately, the CAM preparation consists of a mix of different molecules, often including alkali, with different potential mechanisms of action and, even when favorable results are reported, the role of the single molecules cannot be assessed. Despite all these concerns, CAM products remain quite popular among kidney stone patients. The scarce knowledge in this field prevents one from recommending CAM products in daily clinical practice; only a weak suggestion for their use in kidney stone patients may be reasonable.

## 1. Introduction

Kidney stone disease is frequent in high-income countries, and in the United States and Europe, the prevalence reaches up to 10%. In addition, it shows high rates of recurrences. Of consequence, primary and secondary prevention of kidney stone disease is a relevant task. The medical approach to kidney stone disease is mainly targeted to reduce the risk of stone recurrence [1]. It consists of pharmacological and dietary intervention, but complementary and alternative medicine (CAM) is also often implemented. 

It is known that obesity and metabolic syndrome are associated with urinary stone formation, but there is no similar evidence about total energy intake and nephrolithiasis risk. Hence, kidney stone formers must modulate their energy intake in order to prevent or correct overweight or obesity, but it is the composition of the diet that can modulate the risk of stone formation. Namely, diets rich in fruits and vegetables show an inverse association with the risk of new stone formation because they supply an alkali load that counteracts the net acid generation [2]. Healthy dietary patterns, such as the Dietary Approaches to Stop Hypertension (DASH) or the Mediterranean diet, which are rich in plant foods but not in salt, are associated with a lower prevalence of kidney stone formation compared to the Western diet. In fact, reductions in sodium intake contribute to a reduced risk of kidney stone formation, mainly because of the urine calcium lowering effect. A high intake of animal protein can increase the risk of kidney stones because of the associated increase in urinary calcium and reduction in urinary citrate. With respect to meat proteins, dairy proteins are associated with higher calcium excretion but also increased citrate excretion, and with lower urine excretion of oxalate and uric acid. A high intake of simple carbohydrates can increase urinary calcium, while changes in lipid dietary content do not cause proven changes to urine composition. However, the use of DHA and EPA lipids seems to promote hypercalciuria and hyperoxaluria [3]. The most common, non-pharmacological tool for the prevention of any kind of kidney stones, independently of composition, remains fluid therapy [4]. Fluid intake was found to be inversely related to the risk of kidney stones, with a relative risk of 0.71 in men and of 0.61–0.68 in women [5,6]. A large intake of water is effective in reducing the risk of stone recurrences, provided that it induces a urine volume greater than 2 L per day. Usually, fluid prescription consists of 30 mL/Kg/d. Branded water with not-so-low calcium content (i.e., 40–60 mg/L) is the preferable solution. High drinking levels of sugar-sweetened soda, especially when containing fructose, were associated with a greater prevalence of kidney stone formation [7].

Thus, dietary and lifestyle changes represent an important strategy for the prevention of kidney stone recurrences and cardiovascular damage [7]. A decalogue summarizing general health counseling to kidney stone patients was reported by D’Alessandro et al. as follows: maintain urinary volume over 2 L/day; limit salt intake to 6 g/day; limit animal protein intake; prefer proteins from vegetable sources; do not avoid milk, yogurt, and fresh cheeses; consume plant foods avoiding foods with high oxalate content; reduce/do not increase fat body mass; limit the intake of simple sugars, cholesterol, and saturated fats; prefer complex carbohydrates and olive oil; promote regular physical activity [7].

While the efficacy of dietary changes is effective in reducing lithogenic risk and it is confirmed by epidemiological and physio-pathological studies, little is known about the efficacy and the composition of CAM commercially available products.

## 2. Complementary and Alternative Medicine

When a non-mainstream practice is used together with conventional medicine, it is considered “complementary” and when it is used instead of conventional medicine, it is considered “alternative”.

**Figure 1:**
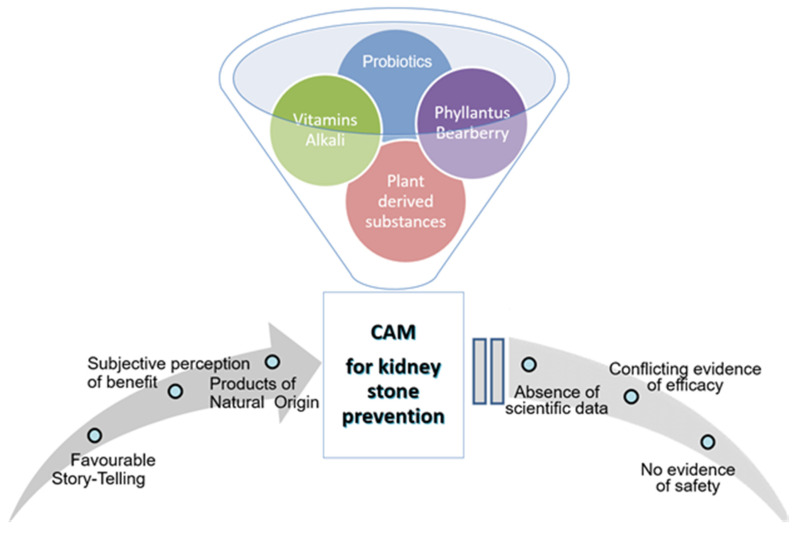
Complementary and Alternative Medicine (CAM) for kidney stone prevention: reasons for common use in presence of poor evidence-based recommendations.

Several patients with kidney stones try to treat their symptoms or to reduce recurrence risk with CAM. One reason for the use of CAM in the kidney stone population is the difficulty in maintaining dietary changes, the side effect profiles of medications, the low cost, and a belief in the safety of CAM preparations [4]. Koo et al. reported that about two-thirds of dietary supplements used to treat or prevent kidney stones have conflicting or insufficient scientific evidence to support their claims [8]. In other words, for the majority of ingredients, there are inadequate or absent data to formulate any evidence-based recommendations (Figure 1). In addition, if any, most of the studies were not on humans, but were on animals and in ex vivo studies.

In a prospective study, Joshi et al. investigated the knowledge and the use of CAM in 103 kidney stone patients, mostly with recurrent disease, by using structured interviews. It emerged that 62% of recurrent kidney stone formers used to take dietary supplements for conditions other than kidney stones, such as multivitamin products (23%) and vitamin D agents (20%), and in 38% of cases, an alkalizing agent was included [4].

Green et al. assessed the knowledge, use, and perceptions of CAM for kidney stones in a diverse, urban population. This cross-sectional study involved 113 patients treated for kidney stones in the Bronx, NY. Knowledge and use of CAM for kidney stones were quite common in the entire cohort, with 44% of subjects having tried CAM for kidney stones. Recurrent stone formers were more likely to have used CAM (first time 30% vs. recurrent 56%, *p* = 0.01). Further, 56% of the reported CAM products were ingredients available from a grocery store: 6% were juices, 28% were teas, 18% were loose herbs, 14% were pills, and 4% were powders. Moreover, 44% of subjects who had used CAM reported symptom improvement, 22% referred to a reduction in kidney stones due to CAM, and 34% considered CAM “very helpful” for kidney stones. However, 8% of those who tried CAM for kidney stones reported an adverse side effect [9].

CAM formulations include pills, powders, drinks, or juices. Patients also use vitamins, pills, and tablet-based supplements, some of which contain alkalis comparable to standard pharmaceutical alkali doses. Apart from alkalis, CAM preparations include vitamins and minerals (vitamin D, vitamin B6, vitamin C, calcium), herbal products, or probiotics.

**Figure 2:**
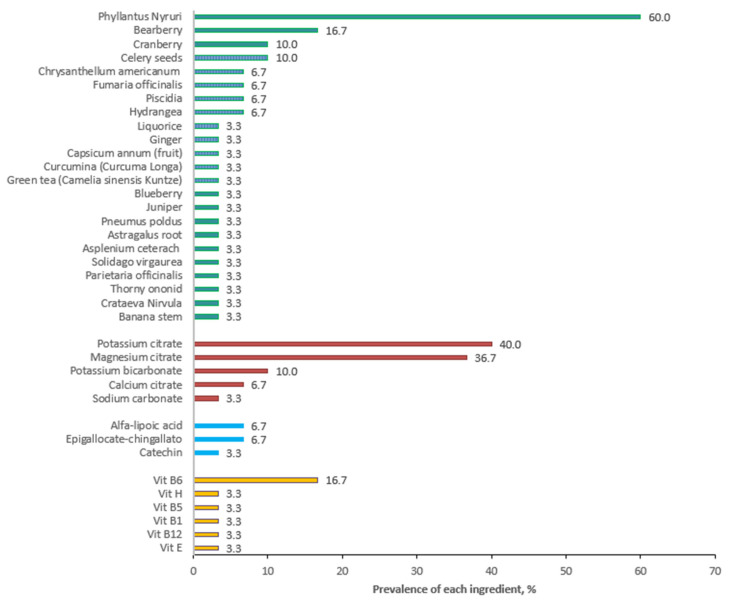
Prevalence of various ingredients in 30 products available on the market and claiming to treat kidney stones. Data are expressed as a percentage.

**Figure 3:**
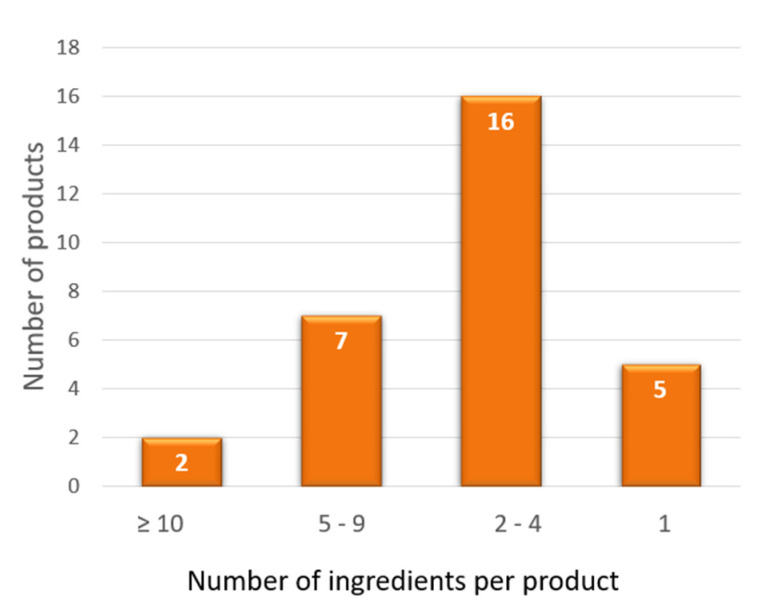
Number of ingredients per product contained in 30 preparations available on the market and claiming to treat kidney stones. The graph shows that the number of ingredients is variable, from only 1 to 16 components.

Figure 2 and Figure 3 summarize the results of our personal observations, obtained from the analysis of 30 preparations available on the market with the indication of kidney stone treatment.

*Phillantus niruri* is the most frequent ingredient, followed, in a smaller percentage, by bearberry and cranberry. Figure 1 shows the presence of other herbal products, but their use is more sporadic.

As regards the alkalizing agents, potassium citrate and magnesium citrate are the most used, as expected, and polyphenols are present in only two of the analyzed products.

As far as vitamins are concerned, as expected, vitamin B6 is the one that appears most frequently.

Further, 33% of the analyzed products present an association with an alkalizing agent (mainly potassium citrate, magnesium citrate). Three of the analyzed supplements contain probiotics: one of the products contains *Lactobacillus casei*, *Lactobacillus plantarum,* and *Bifidobacterium brevis,* associated with alkalizing agents, while the second contains *Enterococcus faecis,* associated with fructo-oligosaccharides (acting as prebiotics) and polyphenols, and a third product generically indicates the presence of five probiotic strains but without defining species and genus. Polyphenols are present to a less extent; the most frequent are catechin and epigallocatechin-3-gallate.

Among drinks, the most used are cranberry juice, lemon juice, apple cider vinegar, and coconut water.

Citrus juices, in particular lemon juice, are sometimes recommended for hypocitraturic patients, although the efficacy of citrus juice supplementation may be mitigated by its sugar content [10,11].

Citrus fruit may be a source of citrate, exerting a favorable effect on calcium stone prevention, but it also contains ascorbic acid that, on the contrary, may lead to elevated oxalate production and urine excretion. Orange juice contains much more ascorbic acid than lemon juice at the same citrate level.

In some papers, an effect of fresh lemon juice supplementation on the prevention of stone recurrence has been hypothesized and studied [12]. A recent Italian randomized controlled study investigated this topic, showing that 60 mL of fresh lemon juice twice daily is similarly effective in reducing the risk, like a standard diet without lemon juice supplementation, during two years of follow-up. Unfortunately, adherence to lemon juice supplementation was 68% at one-year and 48% at two-year follow-up, mainly due to frequent gastrointestinal disorders [13]. This prevented researchers from drawing definitive conclusions, other than those of low palatability and gastrointestinal tolerance to the intervention. These data could suggest that the protective effect of lemon juice supplementation could be explained by a citrate-induced reduction in urinary sodium excretion [14].

Apple cider vinegar contains acetic acid [15] that, after being ingested, exerts an alkalizing effect that protects one from calcium oxalate, cystine, and uric acid stone formation.

Zeng et al. found that people who consumed fermented vinegar (apple cider vinegar) had less chance of developing kidney stones [16].

Apple cider vinegar and other fruit vinegars, such as pomegranate and prickly pear vinegars, are also rich in polyphenols, having higher antioxidant and anti-inflammatory effects [15].

Ghandi et al. evaluated the hypothesis of a protective/prophylactic effect of coconut water in an experimentally induced nephrolithiasis rat model. They observed that treatment with coconut water inhibited crystal deposition in renal tissue and reduced the number of crystals in urine. Furthermore, coconut water also showed a reduced risk of oxidative stress development in the kidney, thus, exerting a protective effect on renal function. The results show a potential role for coconut water in the prevention and treatment of urolithiasis [17].

## 3. Vitamins

Studies about the possible role of vitamins on kidney stone formation have been focused mainly on Vitamin D. This topic was recently reviewed by Bargagli et al. [18] Data from studies of kidney stone primary prevention demonstrate that avoiding low-calcium dietary consumption protects against the risk for calcium kidney stones because adequate calcium intake reduces oxalate intestinal absorption and its urinary concentration. However, calcium supplementation, when assumed between meals, increases urinary calcium excretion, with a negligible effect on urinary oxalate, finally increasing the risk of kidney stones. Regarding vitamin D molecules, active vitamin D is higher in subjects with kidney stones than in subjects without kidney stone history, and 25-hydroxycholecalciferol serum levels are generally higher in kidney stone formers with hypercalciuria. The association between nutritional vitamin D supplements and the risk for stone formation are currently not completely understood. In a review by Bargagli et al., the authors concluded that vitamin D administration may increase the risk for kidney stone formation in patients with hypercalciuria [18]. Of consequence, measuring urine calcium excretion before and after vitamin D, with or without calcium supplementation, is recommended to manage the risk of calcium kidney stone formation [18].

Other vitamins of interest in the field of kidney stone disease are vitamin C and B6 because both are implicated in oxalate metabolism [2].

The overall results suggested that high intake of vitamin C can be a risk factor for kidney stones in males, whereas high dietary vitamin B6 intake seems to exert a protective effect, but only in women [19].

Many studies found that ascorbic acid increases the risk of calcium oxalate stones because it is metabolized directly into oxalate and finally excreted in urine [20]. In fact, Vitamin C supplementation resulted in increased urinary oxalate levels, so ascorbic acid is a risk factor for individuals predisposed to kidney stone production. Data from the Nurses’ Health Study I and II and in the Health Professionals Follow-up Study showed that an intake of total Vitamin C greater than 1000 mg/d was associated with a 41% (95% CI, 11–80%) increased risk for developing a first kidney stone, after multivariate adjustment for other risk factors [5,21,22].

A dose–response relationship was suggested, with HRs of 1.66 for intakes of less than seven vitamin C capsules per day and of 2.23 for intakes of seven or more vitamin C capsules per day [23].

Moreover, vitamin C supplementation at daily dosages of both 250–499 mg and 1000–1499 mg was associated with an 11–14% increased risk of kidney stones. It is remarkable that it occurs in males but not in females [20,23]. Differences by gender are present also for Vitamin B6 intake. 

Vitamin B6 is a cofactor of oxalate metabolism, and this is the reason why it is used in the treatment of primary hyperoxaluria type 1 [19]. A population study showed that Vitamin B6 intake with the diet showed no association with an increased risk of kidney stone formation in males [21]. On the contrary, a high amount of vitamin B6 intake was found to be a protective factor in women, since it was associated with a reduced risk for kidney stone formation [21]. These conclusions were drawn from data in a cohort of 85,557 women with no history of kidney stones, where 1078 incident cases of kidney stones were documented during the 14-year follow-up observation period; with respect to the subjects in the lowest quartile of vitamin B6 intake, those in the highest quartile showed a 34% reduction for the risk of incident kidney stones [21].

## 4. Herbal Products 

Several dietary plant products are used to prevent and manage kidney stone disease, but evidence about their efficacy is still lacking [24].

*Phyllanthus niruri* has mainly litholytic activity, but it also has an antispasmodic, decongestant, antibacterial, and diuretic action. Furthermore, it can prevent the enlargement of stones caused by a continuous deposition of crystals on the surface thanks to a greater release of glycosaminoglycans that create a protein film that surrounds it [25]. *Phyllanthus niruri* was found to decrease urinary oxalate and uric acid in patients with hyperoxaluria and hyperuricosuria, together with an increase in magnesium and potassium urinary excretion [26].

*Chrysanthellum americanum* is another herbal product generally used for its beneficial effect on microcirculation. Chrysantellum extract contributes to increased capillary permeability, induces mild hypotensive activity, and acts favorably on the vascular wall, in the same way as vitamin P. In addition, some recent experimental evidence demonstrated the effect of *Chrysanthellum americanum* in attenuating oxidative stress status in a Rat Model of Irritable Bowel Syndrome [27].

These actions are exerted by flavonoids and saponosides, which Chrysanthellum is rich in. The plant contains maritimeine, mareine, and chrysantellines A and B, the most active molecules, mainly responsible for the effects observed on the microcirculation, although they all act simultaneously. Saponins (surfactant factors), in fact, play a specific role, facilitating the passage of flavonoids through the cell membrane [28,29,30].

Chrysanthellum was found to be effective also in reducing stone formation, thanks to chrysantellin, which inhibits the crystal aggregation process via a mechanism described above [31].

A prospective non-controlled study investigated the effects of a supplement containing *Phyllanthus niruri* and *Chrysanthellum americanum* plus potassium and magnesium citrate in 82 kidney stone patients for 6 months. The formulation included potassium citrate, magnesium citrate, phyllanthus (*Phyllantus niruri*), and chrysanthellum (*Chrysanthellum americanum*) plant. Each patient was also evaluated by computed tomography scan at baseline and at 6 months. Thus, 60.9% showed lower stone dimensions by −6.7 mm ± 3 mm with respect to baseline. At the end of the follow-up period, 27 patients out of 82 were stone-free (32.9%). Further, 59.7% did not show any symptomatic episodes, resulting in a significant improvement in QoL [32]. In addition to these favorable results, this study demonstrates the efficacy of the preparation, but not of the single molecules.

Bearberry belongs to the Ericacee family. Its use in CAM preparations for kidney stones is probably due to the presence of flavonoids and mainly to its antiseptic activity. The latter depends to the presence of arbutin and methylarbutin, components which are transformed by enzymatic hydrolysis catalyzed by the enzyme arbutase into hydroquinone and methylihydroquinone, substances with bactericidal and anti-adhesive action on pathogens [33].

Goldenrod (*Solidago virgaurea* L.) extract was used for centuries because of its wide and quite complex action spectrum. It has, indeed, an anti-inflammatory, antimicrobial, diuretic, antispasmodic, and analgesic effect and it is recommended, in particular, for the treatment of infections and inflammations, but also in the prevention of kidney stone formation and to help remove urinary gravel. This therapy is considered safe and does not show drug-related side effects [34].

The common name “stonebreaker” usually refers to the plant belonging to the genus Asplenium species ceterach (scientific name *Ceterach officinarum* Willd. or *Asplenium ceterach* L.). Known also as cedracca or russet grass, the stonebreaker has always been considered in popular medicine as a very useful remedy against small kidney stones and inflammatory disorders of the urinary tract.

There is also another type of plant called “stonebreaker”, *Phyllanthus niruri,* as discussed above. The cedracca is a small fern native to the Eurasian continent that grows in regions with a mild climate, but it is also widespread in Italy; it grows on stone and brick walls as well as between rocks. This plant contains phytochemicals of polyphenolic origin, such as flavonol hyperoside and the phenolic acid chlorogenic acid [35]. The latter seems to be responsible for the litholytic action of Ceterach.

Celery seeds aqueous extract and celery seed oil extract have been shown to have anti-inflammatory and anti-oxidant effects in rat models. These properties depend on the content of various types of flavonoids, such as quercetin and taxifolin. Evidence exists for a reduction in serum uric acid levels in mice with hyperuricemia and the swelling rates of ankle joints in rats with gouty arthritis, which may be associated with the modulation of xanthine oxidase activity and a lower inflammation response due to oxidative stress regulation [36]. There is no evidence of a direct effect of celery seed extracts on kidney stone production; thus, the presence of this compound in CAM preparations probably has the aim of treating inflammation and pain due to the presence of stones in the urinary tract.

Cranberry is a popular ingredient in dietary supplements and it is commonly used in the prevention of urinary tract infections. Clinical studies on humans have reported contradictory results about cranberry supplementation and its role in kidney stone formation [37].

Some studies showed that uric acid or calcium oxalate stone formers are at risk when consuming cranberry products [38], while other studies have reported that consumption of large amounts of cranberry may reduce kidney stone formation [39,40]. Data suggesting that kidney stone formation is associated with the intake of cranberry are not sufficient. More studies are required to better clarify the association between cranberry and kidney stone formation, with a clear identification of the bioactive constituents present in cranberry preparations [41].

*Fumaria officinalis*-*fumitory,* or earth smoke, is a medicinal plant used in empirical medicine in numerous countries [42]. There are no placebo-controlled studies but several empirical reports, clinical case reports, and animal experimental studies have been published regarding its antispasmodic and diuretic actions due to the presence of protopin and flavonoids, such as Rutin and Quercetin. For this reason, in Germany, *Fumaria officinalis* is approved for the indication of “colicky pain affecting the gallbladder and biliary system, together with the gastrointestinal tract” [43].

Piscidia (*Piscidia erythrina*) is another ingredient found in CAM supplements for kidney stones. There are no studies explaining a direct involvement of this herb in kidney stone formation/prevention. Piscidia is known for its antispasmodic and analgesic action determined by the presence of rotenone, which exerts a papaverine-like action [44].

This effect could justify its use in nephrolithiasis treatment, even if studies should be implemented to better clarify the role of Piscidia in kidney stone formation/prevention and its possible use, as a consequence.

Unfortunately, most CAM preparations consist of a mix of different molecules (Figure 2), including alkalis, with different potential mechanisms of action, so that the roles of the single components remain uncertain.

## 5. Probiotics

A growing body of evidence indicates the potential role of the intestinal microbiota in the pathogenesis of kidney stone formation [45,46,47]. Gut microbiota seem to affect urine composition, thus, increasing the risk of kidney stone incidence [48].

Recurrent calcium stone patients have lower fecal microbial diversity compared to non-stone formers. The former showed a lower prevalence of oxalate-degrading bacteria than the latter [45,46]. Bacteroides were more prevalent in kidney stone formers, while Prevotella was less represented. *Oxalobacter formigenes* was the most studied intestinal bacterium in patients with urolithiasis [49,50,51].

Gut colonization by *Oxalobacter formigenes* was confirmed to be lower in kidney stone formers than in control subjects, especially in recurrent stone formers [2,51].

This condition can occur after prolonged antibiotic therapy [2]. To confirm, evidence exists of an increased risk of developing kidney stones in people frequently receiving antibiotics [45,52].

*Oxalobacter formigenes* is dependent on oxalate as a unique energy substrate. Thereby, it uses oxalate present in the intestinal milieu, reducing oxalate intestinal absorption and oxalate urine excretion, as well as calcium oxalate urine supersaturation [45,52,53].

Troxel et al., in a prospective, controlled study, found that calcium oxalate stone formers had a low rate of colonization with *Oxalobacter formigenes*. Among stone formers, the absence of intestinal Oxalobacter correlates with higher urinary oxalate concentration and an increased risk of hyperoxaluria [54].

Unfortunately, two trials using *Oxalobacter formigenes* preparations did not confirm the ability to reduce oxaluria [45].

In addition to *Oxalobacter formigenes*, *Lactobacillus* spp. and *Bifidobacterium* spp. are able to degrade dietary oxalate in the gastrointestinal tract, limiting its absorption and, hence, its urine excretion [55,56].

Despite this, Tavasoli et al., in a randomized, placebo-controlled, double-blind, and in-vitro trial, showed that the consumption of a probiotic supplement containing *Lactobacillus acidophilus* and *Bifidobacterium lactis* did not affect urine oxalate [57].

It is noteworthy that *Lactobacillus* spp. and *Bifidobacterium* spp. preparations are generally regarded as safe by the FDA, while Oxalobacter ones are not. This is probably because the effects of Bifidobacterium and Lactobacillus supplementation have been widely investigated, both in vitro and in vivo, with interesting results, as regards their potential positive immunomodulating effect [58,59].

Thus, the use of probiotics may have favorable effects in the treatment of renal stone disease, as well as for other diseases [60]. Probiotic preparations including oxalate-degrading bacteria could be useful in decreasing urinary oxalate excretion and in calcium oxalate stone prevention. In any case, further investigation in long-term studies in various patient populations is needed [61].

Unfortunately, it is not easily included in commercial preparations [45]. The difficulty in making probiotics containing *Oxalobacter formigenes* may be due to its sensitivity to oxygen and low pH, the strict oxalate demand for growth, and intolerance to the processes, which are commonly involved in the manufacture, storage, and distribution of probiotic products.

The use of a mixture containing Lactobacillus or Bifidobacterium, and bacteria with different rates of oxalate-metabolizing capacity, may contribute to the degradation of oxalate and the reduction in its excretion in urine [45,47,62,63].

## 6. Conclusions

Most dietary supplements claiming to treat or prevent kidney stones contain ingredients with conflicting or no scientific evidence to support their claims. Clinicians should advise stone formers that the effects of most supplements are unknown or unstudied in humans, and that the absence of evidence does not imply absence of potential harm.

Unfortunately, CAM preparations consist of a mix of different molecules, often including alkalis, with different potential mechanisms of action and, even when favorable results are reported, the role of single molecules cannot be assessed. Despite all these concerns, CAM preparation products remain quite popular among the population of kidney stone patients (Figure 1). 

As a whole, the scarce knowledge in this field prevents one from recommending CAM preparation products in daily clinical practice; only a weak suggestion for their use is reasonable.

## Data Availability

Not applicable.

## References

[1] Siener R. (2021). Nutrition and Kidney Stone Disease. Nutrients.

[2] Pedro R.N., Aslam A.U., Bello J.-O.-, Bhatti K.H., Philipraj J., Sissoko I., Vasconcellos G.S., Trinchieri A., Buchholz N. (2020). Nutrients, vitamins, probiotics and herbal products: An update of their role in urolithogenesis. Urolithiasis.

[3] Taylor E.N., Stampfer M.J., Curhan G.C. (2005). Fatty acid intake and incident nephrolithiasis. Am. J. Kidney Dis..

[4] Joshi A., Tallman J.E., Calvert J.K., Brewer T., Miller N.L., Yang L., Asplin J.R., Hsi R.S. (2021). Complementary and Alternative Medicine Use in First-time and Recurrent Kidney Stone Formers. Urology.

[5] Curhan G.C., Willett W.C., Knight E.L., Stampfer M.J. (2004). Dietary factors and the risk of incident kidney stones in younger women (Nurses Health Study II). Arch. Intern. Med..

[6] Curhan G., Willett W., Speizer F., Spiegelman D., Stampfer M.J. (1997). Comparison of dietary calcium with supplemental calcium and other nutrients as factors affecting the risk for kidney stones in women. Ann. InternMed..

[7] D’Alessandro C., Ferraro P.M., Cianchi C., Barsotti M., Gambaro G., Cupisti A. (2019). Which Diet for Calcium Stone Patients: A Real-World Approach to Preventive Care. Nutrients.

[8] Koo K., Aro T., Matlaga B.R. (2020). Buyer Beware: Evidence-Based Evaluation of Dietary Supplements for Nephrolithiasis. J. Endourol..

[9] Green B., Feiertag N., Watts K.L., Small A.C. (2022). Evaluating perceptions and usage of natural remedies, herbal medicine, and dietary supplements for kidney stones among a diverse, international, urban patient population. Urolithiasis.

[10] Zuckerman J.M., Assimos D.G. (2009). Hypocitraturia: Pathophysiology and medical management. Rev. Urol..

[11] Kang D.E., Sur R.L., Haleblian G.E., Fitzsimons N.J., Borawski K.M., Preminger G.M. (2007). Long-term lemonade based dietary manipulation in patients with hypocitraturic nephrolithiasis. J. Urol..

[12] Penniston K.L., Steele T.H., Nakada S.Y. (2007). Lemonade therapy increases urinary citrate and urine volumes in patients with recurrent calcium oxalate stone formation. Urology.

[13] Ruggenenti P., Caruso M.R., Cortinovis M., Perna A., Peracchi T., Giuliano G.A., Rota S., Brambilla P., Invernici G., Villa D. (2021). Fresh lemon juice supplementation for the prevention of recurrent stones in calcium oxalate nephrolithiasis: A pragmatic, prospective, randomized, open, blinded endpoint (PROBE) trial. EClinicalMedicine.

[14] Durland J., Schumann S.O. (2022). A Rocky Discontinuation of Diet Mountain Dew. J. Investig. Med. High Impact Case Rep..

[15] Bouazza A., Bitam A., Amiali M., Bounihi A., Yargui L., Koceir E.A. (2016). Effect of fruit vinegars on liver damage and oxidative stress in high-fat-fed rats. Pharm. Biol..

[16] Zeng G., Mai Z., Xia S., Wang Z., Zhang K., Wang L., Long Y., Ma J., Li Y., Wan S.P. (2017). Prevalence of kidney stones in China: An ultrasonography based cross-sectional study. BJU Int..

[17] Gandhi M., Aggarwal M., Puri S., Singla S.K. (2013). Prophylactic effect of coconut water (*Cocos nucifera* L.) on ethylene glycol induced nephrocalcinosis in male wistar rat. Int. Braz. J. Urol..

[18] Bargagli M., Ferraro P.M., Vittori M., Lombardi G., Gambaro G., Somani B. (2021). Calcium and Vitamin D Supplementation and Their Association with Kidney Stone Disease: A Narrative Review. Nutrients.

[19] Curhan G.C., Willett W.C., Rimm E.B., Stampfer M.J. (1996). A prospective study of the intake of vitamins C and B6, and the risk of kidney stones in men. J. Urol..

[20] Jiang K., Tang K., Liu H., Xu H., Ye Z., Chen Z. (2019). Ascorbic Acid Supplements and Kidney Stones Incidence Among Men and Women: A systematic review and meta-analysis. Urol. J..

[21] Curhan G.C., Willett W.C., Speizer F.E., Stampfer M.J. (1999). Intake of vitamins B6 and C and the risk of kidney stones in women. JASN.

[22] Taylor E.N., Stampfer M.J., Curhan G.C. (2004). Dietary factors and the risk of incident kidney stones in men: New insights after 14 years of follow-up. J. Am. Soc. Nephrol. JASN.

[23] Ferraro P.M., Curhan G.C., Gambaro G., Taylor E.N. (2016). Total, Dietary, and Supplemental Vitamin C Intake and Risk of Incident Kidney Stones. Am. J. Kidney Dis..

[24] Nirumand M.C., Hajialyani M., Rahimi R., Farzaei M.H., Zingue S., Nabavi S.M., Bishayee A. (2018). Dietary Plants for the Prevention and Management of Kidney Stones: Preclinical and Clinical Evidence and Molecular Mechanisms. Int. J. Mol. Sci..

[25] Freitas A.M., Schor N., Boim M.A. (2002). The effect of Phyllanthus niruri on urinary inhibitors of calcium oxalate crystallization and other factors associated with renal stone formation. BJU Int..

[26] Pucci N.D., Marchini G.S., Mazzucchi E., Reis S.T., Srougi M., Evazian D., Nahas W.C. (2018). Effect of phyllanthus niruri on metabolic parameters of patients with kidney stone: A perspective for disease prevention. Int. Braz. J. Urol..

[27] Cojocariu R., Ciobica A., Balmus I.M., Guenne S., Trifan A., Stanciu C., Hrițcu L., Lefter R. (2019). Antioxidant Capacity and Behavioral Relevance of a Polyphenolic Extract of Chrysanthellum americanum in a Rat Model of Irritable Bowel Syndrome. Oxid. Med. Cell Longev..

[28] Amos S., Binda L., Adamu M., Vongtau H.O., Abbah J., Omogbai E.K., Akah P.A., Bukar B.B., Wambebe C., Gamaniel K. (2003). Effect of the aqueous extract of Chrysanthellum indicum on calcium mobilization and activation of rat portal vein. J. Ethnopharmacol..

[29] Lievre H., Guillot B. (1983). Le Chrysanthellum americanum. Rev. Jeune Med..

[30] Ghédira K., Goetz P. (2017). Chrysanthellum: *Chrysanthellum americanum* (L.) Vatke (*Asteraceae*). Phytothérapie.

[31] Becchi M., Bruneteau M., Trouilloud M., Combier H., Sartre J., Michel G. (1979). Structure of a new saponin: Chrysantellin A from *Chrysanthellum procumbens* Rich. Eur. J. Biochem..

[32] Cai T., Tiscione D., Puglisi M., Malossini G., Ruggera L., Verze P., Arcaniolo D., Palmieri A. (2021). Phyllanthus niruri and Chrysanthellum americanum in association with potassium and magnesium citrates are able to prevent symptomatic episode in patients affected by recurrent urinary stones: A prospective study. Arch. Ital. Urol. Androl..

[33] Grases F., Melero G., Costa-Bauzá A., Prieto R., March J.G. (1994). Urolithiasis and phytotherapy. Int. Urol. Nephrol..

[34] Melzig M.F. (2004). Echtes Goldrutenkraut—Ein Klassiker in der urologischen Phytotherapie [Goldenrod—A classical exponent in the urological phytotherapy]. Wien. Med. Wochenschr..

[35] Farràs A., Mitjans M., Maggi F., Caprioli G., Vinardell M.P., López V. (2022). Exploring wild Aspleniaceae ferns as safety sources of polyphenols: The case of *Asplenium trichomanes* L. and *Ceterach officinarum* Willd. Front Nutr..

[36] Li S., Li L., Yan H., Jiang X., Hu W., Han N., Wang D. (2019). Anti-gouty arthritis and anti-hyperuricemia properties of celery seed extracts in rodent models. Mol. Med. Rep..

[37] Gardner Z., McGuffin M. (2013). American Herbal Products Association’s Botanical Safety Handbook.

[38] Terris M.K., Issa M.M., Tacker J.R. (2001). Dietary supplementation with cranberry concentrate tablets may increase the risk of nephrolithiasis. Urology.

[39] American Botanical Council (ABC) Clinical Guide to Herbs. Cranberry Monograph. http://cms.herbalgram.org/ABCGuide/Monographs/Cranberry.html.

[40] McHarg T., Rodgers A., Charlton K. (2003). Influence of cranberry juice on the urinary risk factors for calcium oxalate kidney stone formation. BJU Int..

[41] Madden E., McLachlan C., Oketch-Rabah H., Calderón A.I. (2021). Safety of Cranberry: Evaluation of Evidence of Kidney Stone Formation and Botanical Drug- Interactions. Planta Med..

[42] Gupta P.C., Sharma N., Rao C.V. (2012). A review on ethnobotany, phytochemistry and pharmacology of Fumaria indica (Fumitory). Asian Pac. J. Trop Biomed..

[43] Hentschel C., Dressler S., Hahn E.G. (1995). Fumaria officinalis (Echter Erdrauch)—Klinische Anwendung [Fumaria officinalis (fumitory)—Clinical applications]. Fortschr. Med..

[44] Della Loggia R., Zilli C., Del Negro P., Redaelli C., Tubaro A. (1988). Isoflavones as spasmolytic principles of Piscidia erythrina. Prog. Clin. Biol. Res..

[45] Wigner P., Bijak M., Saluk-Bijak J. (2022). Probiotics in the Prevention of the Calcium Oxalate Urolithiasis. Cells.

[46] Stern J.M., Moazami S., Qiu Y., Kurland I., Chen Z., Agalliu I., Burk R., Davies K.P. (2016). Evidence for a distinct gut microbiome in kidney stone formers compared to non-stone formers. Urolithiasis.

[47] Sharma A.P., Burton J., Filler G., Dave S. (2020). Current update and future directions on gut microbiome and nephrolithiasis. Indian J. Urol..

[48] Mehta M., Goldfarb D.S., Nazzal L. (2016). The role of the microbiome in kidney stone formation. Int. J. Surg..

[49] Allison M.J., Dawson K.A., Mayberry W.R., Foss J.G. (1985). Oxalobacter formigenes gen. nov., sp. nov.: Oxalate-degrading anaerobes that inhabit the gastrointestinal tract. Arch. Microbiol..

[50] Allison M.J., Cook H.M., Milne D.B., Gallagher S., Clayman R.V. (1986). Oxalate degradation by gastrointestinal bacteria from humans. J. Nutr..

[51] Abratt V.R., Reid S.J. (2010). Oxalate-degrading bacteria of the human gut as probiotics in the management of kidney stone disease. Adv. Appl. Microbiol..

[52] Liu M., Nazzal L. (2019). Enteric hyperoxaluria: Role of microbiota and antibiotics. Curr. Opin. Nephrol. Hypertens..

[53] Knight J., Deora R., Assimos D.G., Holmes R.P. (2013). The genetic composition of Oxalobacter formigenes and its relationship to colonization and calcium oxalate stone disease. Urolithiasis.

[54] Troxel S.A., Sidhu H., Kaul P., Low R.K. (2003). Intestinal Oxalobacter formigenes colonization in calcium oxalate stone formers and its relation to urinary oxalate. J. Endourol..

[55] Murphy C., Murphy S., O’Brien F., O’Donoghue M., Boileau T., Sunvold G., Reinhart G., Kiely B., Shanahan F., O’Mahony L. (2009). Metabolic activity of probiotics-oxalate degradation. Vet. Microbiol..

[56] Mogna L., Pane M., Nicola S., Raiteri E. (2014). Screening of different probiotic strains for their in vitro ability to metabolise oxalates: Any prospective use in humans?. J. Clin. Gastroenterol..

[57] Tavasoli S., Jalali S., Naji M., Borumandnia N., Shakiba Majd G., Basiri A., Khosravi Darani K., Karamad D., Tajabadi-Ebrahimi M., Taheri M. (2021). Effect of a Probiotic Supplement Containing Lactobacillus Acidophilus and Bifidobacterium Animalis Lactis on Urine Oxalate in Calcium Stone Formers with Hyperoxaluria: A Randomized, Placebo-controlled, Double-blind and In-vitro Trial. Urol. J..

[58] Christensen H.R., Larsen C.N., Kaestel P., Rosholm L.B., Sternberg C., Michaelsen K.F., Frøkiaer H. (2006). Immunomodulating potential of supplementation with probiotics: A dose-response study in healthy young adults. FEMS Immunol. Med. Microbiol..

[59] Ashraf R., Shah N.P. (2014). Immune system stimulation by probiotic microorganisms. Crit. Rev. Food Sci. Nutr..

[60] Lieske J.C. (2017). Probiotics for prevention of urinary stones. Ann. Transl. Med..

[61] Hatch M., Cornelius J., Allison M., Sidhu H., Peck A., Freel R.W. (2006). *Oxalobacter* sp. reduces urinary oxalate excretion by promoting enteric oxalate secretion. Kidney Int..

[62] Siva S., Barrack E.R., Reddy G.P., Thamilselvan V., Thamilselvan S., Menon M., Bhandari M. (2009). A critical analysis of the role of gut Oxalobacter formigenes in oxalate stone disease. BJU Int..

[63] Karamad D., Khosravi-Darani K., Khaneghah A.M., Miller A.W. (2022). Probiotic Oxalate-Degrading Bacteria: New Insight of Environmental Variables and Expression of the oxc and frc Genes on Oxalate Degradation Activity. Foods.

